# Vaccination with Tozimameran Induces T-Cell Activation, but Not Senescent or Exhaustive Alterations, in Kidney Transplant Recipients

**DOI:** 10.3390/vaccines12080877

**Published:** 2024-08-02

**Authors:** Stamatia Stai, Georgios Lioulios, Aliki Xochelli, Anastasia Papadopoulou, Evangelia Yannaki, Efstratios Kasimatis, Michalis Christodoulou, Eleni Moysidou, Margarita Samali, Theodolinda Testa, Artemis Maria Iosifidou, Myrto Aikaterini Iosifidou, Georgios Tsoulfas, Maria Stangou, Asimina Fylaktou

**Affiliations:** 1 School of Medicine, Aristotle University of Thessaloniki, 54636 Thessaloniki, Greece; staimatina@yahoo.gr (S.S.); pter43@yahoo.gr (G.L.); michalischristodoulou22@gmail.com (M.C.); moysidoueleni@yahoo.com (E.M.); ios.artemis.2000@gmail.com (A.M.I.); ios.myrtv.2002@gmail.com (M.A.I.); 2 1st Department of Nephrology, Hippokration General Hospital, 54642 Thessaloniki, Greece; frasci@outlook.com.gr; 3 Department of Immunology, National Histocompatibility Center, Hippokration General Hospital, 54642 Thessaloniki, Greece; aliki.xochelli@gmail.com (A.X.); margaritasamali@gmail.com (M.S.); fylaktoumina@gmail.com (A.F.); 4 Hematology Department, Hematopoietic Cell Transplantation Unit, Gene and Cell Therapy Center, George Papanikolaou Hospital, 57010 Thessaloniki, Greece; eyannaki@uw.edu; 5 Department of Transplant Surgery, Hippokration General Hospital, 54642 Thessaloniki, Greece; tsoulfasg@auth.gr

**Keywords:** kidney transplant recipients, SARS-CoV-2, tozinameran, immunosenescence, immunoexhaustion

## Abstract

Background: Multiple vaccinations have potential inimical effects on the immune system aging process. We examined whether response to SARS-CoV-2 vaccination with Tozinameran is associated with immunosenescence and immunoexhaustion in kidney transplant recipients (KTRs). Methods: In this prospective observational study, we observed 39 adult kidney transplant recipients (KTRs) who had no pre-existing anti-SARS-CoV-2 antibodies and were on stable immunosuppression. CD4+ and CD8+ T-cell subpopulations [comprising CD45RA+CCR7+ (naïve), CD45RA−CCR7+ (T-central memory, TCM), CD45RA−CCR7− (T-effector memory, TEM) and CD45RA+CCR7− (T-effector memory re-expressing CD45RA, T_EMRA_, senescent), CD28− (senescent) and PD1+ (exhausted)] were evaluated at 2 time points: T1 (48 h prior to the 3rd), and T2 (3 weeks following the 3rd Tozinameran dose administration). At each time point, patients were separated into Humoral and/or Cellular Responders and Non-Responders. Results: From T1 to T2, CD4+TCM and CD8+TEM were increased, while naïve CD4+ and CD8+ proportions were reduced in the whole cohort of patients, more prominently among responders. At T2, responders compared to non-responders had higher CD8+CD28+ [227.15 (166) vs. 131.44 (121) cells/µL, *p*: 0.036], lower CD4+CD28− T-lymphocyte numbers [59.65 (66) cells/µL vs. 161.19 (92) cells/µL, *p*: 0.026] and percentages [6.1 (5.5)% vs. 20.7 (25)%, *p*: 0.04]. Conclusion: In KTRs, response to vaccination is not associated with an expansion of senescent and exhausted T-cell concentrations, but rather with a switch from naïve to differentiated-activated T-cell forms.

## 1. Introduction

Immune senescence and immune exhaustion are two distinct functional states. Senescence is a result of impaired telomerase activity and subsequent telomere length decrease, emerging in the course of cellular differentiation process. It is characterized by irreversible loss of proliferating capacity and concurrent increased secretion of pro-inflammatory cytokines after stimulation. The differentiation status of T-lymphocytes can be determined based on the presence of certain surface molecules, including CD45RA, CD45RO, C-C chemokine receptor 7 (CCR-7), CD57, killer cell lectin-like receptor sub-family G (KLRG-1), CD27 and CD28, with more senescent subsets lacking expression of the latter two, and re-expressing the RA isoform of CD45. On the contrary, immune exhaustion is a reversible condition, associated with other markers, such as programmed cell death 1 (PD-1), lymphocyte activation gene 3 (LAG-3), T-cell immunoglobin mucin 3 (TIM-3) and cytotoxic T-lymphocyte-associated protein 4 (CTLA-4). Exhausted cells are prone to apoptosis, not necessarily highly differentiated and are unable to both produce cytokines with stimulation and proliferate [1].

Apart from accelerated immune senescence (mostly attributed to premature thymic involution), end-stage renal disease (ESRD) is characterized by the presence of low-grade inflammation. The latter results from the combination of somatic cell senescence associated secretory phenotype (SASP), chronic viral infections, oxidative stress, exposure to dialysis materials, microbiota dysbiosis, impaired thymic negative selection of auto-reactive T-cells, reduced regulatory T-cell (Treg) formation, etc. [2]. Immune exhaustion is also observed among chronic kidney disease (CKD) patients and seems to gradually aggravate along with the decline in kidney function [3], a fact that could possibly be explained in the context of a chronic inflammation-mediated stimulation [4]. The co-existence of immune deficiency and inflammation is known as “inflammaging” [2] and can only be partially mitigated after a successful renal transplantation (e.g., through reduction in inflammation due to the use of immunosuppressive agents) [2,5].

Indeed, as indicated by the presence of functional deficits in acquired immunity, as well of divergence in immune cell subpopulations’ composition, the reinstation of a healthy immune cell profile that would replace the end-stage renal disease (ESRD)-associated one, cannot be achieved after renal transplantation [6]. More specifically, kidney transplant recipients (KTRs) present with a lower-than-normal thymic output, characterized by homeostatic proliferation of naïve T-cells [7] and display increased concentrations of senescent lymphocytes [8]. These changes can possibly be attributed to the accumulation of uremia-induced epigenetic changes, that cannot be eliminated after transplantation [7].

As anticipated, the combination of an impaired, “aged” immune system status with chronic immunosuppressive treatment administration, renders KTRs unable to mount immune responses after vaccinations in terms of both humoral and cellular immunity activation. Vaccination against the SARS-CoV-2 virus could not be an exception that to that rule [6,9,10].

Nevertheless, although the negative effect of senescence and exhaustion on vaccine efficacy has been widely studied, not many research works regarding the potential impact of multiple vaccinations on the immune system differentiation and exhaustion status are currently available. Interestingly, based on the findings of relatively recent studies on influenza immunization, several authors have supported that repeated vaccinations could even be associated with suboptimal effectiveness, with reduced antibody-affinity maturation and antigenic distance hypothesis being only two of the possible underlying mechanisms [11]. Other researchers have also proposed that anti-SARS-CoV-2 mRNA vaccine booster dose administration is a potential obstacle regarding the achievement of robust immune responses and perhaps promotes immune exhaustion in certain groups of immunocompromised individuals [12,13]. In that context, clarifying whether this is the case for KTRs is of great importance, as it could affect future decision-making on vaccination strategies.

In the present study, we attempted to examine whether the response to SARS-CoV-2 vaccination with Tozinameran (BNT162b2) could favor the development of immunosenescence and immunoexhaustion among KTRs, focusing on T-cell subpopulations. 

## 2. Materials and Methods

### 2.1. Patients

Our sample consisted of 39 adult (age ≥ 18 years) KTRs, younger than 75 years old, who were being followed in the outpatient clinics of the Aristotle University of Thessaloniki (AUTH) 1st Nephrology Department and Department of Transplant Surgery. They had received a kidney graft at least 3 months prior to their recruitment for the study and were under stable immunosuppressive treatment with a triple combination of a corticosteroid regimen, a calcineurin inhibitor (CNI) and mycophenolic acid (MPA) or mycophenolate mofetil (MMF). All patients had been immunized against the Influenza, Hepatitis B and Varicella Zoster viruses, Pneumoniococcus, *Haemofilus influeznae* and Meningitididoccus based on the program of the Greek Vaccination Committee. They had a body mass index (BMI) of less than 25 and a positive history for arterial hypertension. All of them tested negative for pre-existing antibody (Ab) immunity to coronavirus disease 2019 (COVID-19), as documented by the absence of anti-SARS-CoV-2 antibodies in blood samples selected 48 h before the vaccination schedule initiation. All participants had been thoroughly informed about the study and had signed an informed consent. They followed the immunization program of Greece’s National Vaccination Committee with Tozinameran against the SARS-CoV-2. More specifically, they received the 2nd vaccination dose 3 weeks after the 1st and the 3rd one 16 weeks following the 2nd.

Exclusion criteria were: age < 18 years, the presence of comorbidities (such as active systemic diseases, solid organ or hematologic malignancies) during the 2 years preceding the study initiation, history of bacterial or viral infection in the past trimester, acute humoral or cellular rejection events in during the last 6 months, recent (<2 years before patient recruitment) administration of chemotherapeutic regimens or rituximab.

### 2.2. Schedule of the Study

Our study was prospective observational research. Blood samplings were performed at 2 time points: T1, which was 48 h before, and T2, which was 3 weeks after the 3rd Tozinameran dose administration. At each time point, the following parameters were examined:

(a) Anti-SARS-CoV-2 neutralizing antibody (NAb) levels, utilizing chemiluminescence immunoassay (CLIA)
(b) Viral-specific interferon gamma (IFN-γ) production, measured with enzyme-linked immunosorbent spot (ELISpot)
(c) T-lymphocyte subpopulation concentrations, with the use of flow cytometry.

During the first patient visit, demographic, clinical and laboratory information were recorded.

At each time point (T1, T2), patients were separated into responders and non-responders, with the first ones displaying adequate protective humoral and/or viral-specific T-cellular response (e.g., they had protective NAb levels and/or a positive ELISpot test) and the second ones lacking both of the aforementioned conditions. We compared the differences in several cellular subpopulations’ compositions (concentrations and percentages), defined with regards to the senescence and exhaustion phenomena, between responders and non-responders both at T1 and T2. We also examined whether changes in these compositions in the time interval between T1 and T2 varied between the two patient groups.

### 2.3. Laboratory Methods

#### 2.3.1. Flow Cytometry

Heparinized blood samples were selected from all participants and were processed in order to estimate the concentrations and percentages of several T-lymphocyte subsets. All utilized receptors (CD3, CD4, CD8, CD28, CD45RA, CCR7, PD1) were detected with a cytometer (Navios Flow Cytometer, Beckman Coulter, Brea, CA, USA). The monoclonal antibodies used to recognize membrane receptors were: CD45 PC7, CD3 FITC, CD4 Pacific Blue, CD8 PC5, CD31 ECD, CD45RA APC, CCR7 PE, CD28 ECD.

Based on the expression of the above-mentioned cells surface receptors, the described CD3+CD4+ and CD3+CD8+ cell subpopulations were segregated as:

(i) recent thymic emigrants (CD3+CD4+CD31+ or CD3+CD8+CD31+),
(ii) naïve T-cells (CD3+CD4+CD45RA+CCR7+ or CD3+CD8+CD45RA+CCR7+),
(iii) TCM (CD3+CD4+CD45RA−CCR7+ or CD3+CD8+CD45RA−CCR7+),
(iv) TEM (CD3+CD4+CD45RA−CCR7− or CD3+CD8+CD45RA−CCR7−)
(v) T_EMRA_ (CD3+CD4+CD45RA+CCR7− or CD3+CD8+CD45RA+CCR7−).
(vi) CD3+CD4+CD28+, CD3+CD8+CD28+ and CD3+CD4+CD28−, CD3+CD8+CD28−
(vii) CD3+CD4+PD1+, CD3+CD8+PD1+

T_EMRA_ and CD28− cells were regarded as senescent, while the presence of PD1 molecule was considered an indicator of exhaustion.

The gating strategy used and definition of T-cell subsets is shown in Supplement Figures S1 and S2.

#### 2.3.2. Chemiluminescence Immunoassay

A direct CLIA assay [Maglumi™ 2000 Plus (New Industries Biomedical EngineeringCo., Ltd. (Snibe), Shenzhen, China)] was used for the measurement of anti-SARS-CoV-2 IgG NAb titers. Magnetic microbeads coated with angiotensin-converting enzyme 2 (ACE2) antigen and N-(4-Amino-Butyl)-N-Ethyl-Isoluminol (ABEI) labeled with recombinant S protein receptor binding domain (S-RBD) antigen were intubated with the samples. NAbs present in the samples competed with the ACE2 antigens (which were immobilized on the magnetic microbeads) for the binding recombinant ABEI-labeled S-RBD antigen. The light signal was measured as relative light units (RLUs), with the result being inversely proportional to the concentration of the anti-SARS-CoV-2 Nabs. 

According to the manufacturer, NAb concentrations ≥ the cut-off lever of 0.3 AU/mL, were considered protective.

#### 2.3.3. Enzyme-Linked Immunosorbent Spot

Peripheral blood mononuclear cells (PBMCs) were stimulated with overlapping 15-mer Pep-Mixes of the full-length spike protein (JPT Peptide Technologies). ELISpot was utilized for the measurement of IFN-γ, which was produced by viral-specific T-cells upon stimulation. Spot-forming cells (SFCs) were counted using an Eli.Scan ELISpot scanner (A.EL.VIS) running the Eli.Analyse software V6.2.SFC. SARS-CoV-2 spike-specific T-cells were expressed as SFCs per input cells. The response was considered positive if the total cytokine-producing SFCs against spikes were ≥30 per 5 × 10^5^ PBMCs.

### 2.4. Statistical Analysis

The IBM SPSS 26.0 (SPSS Inc., Chicago, IL, USA) program was utilized for statistical analyses, with the results having a *p* value of <0.05 being considered statistically significant. We examined all quantitative variables for the presence of normal distribution with the use of the Shapiro–Wilk test. Since all of them followed a non-normal distribution, the central tendency measure we preferred was the median value (interquartile range, IQR). Pairwise comparisons for two median values of successive results of parameters under study were performed with the Wilcoxon test, while the Mann–Whitney U test was used in order to compare the mean vales of two independent groups. 

## 3. Results

### 3.1. Patient Characteristics 

The median age of our patients was 47 (16) years, they had received a kidney graft 6.9 (14.5) years prior to the study initiation and their median eGFR value was 54.1 (22.7) mL/min/1.73 m^2^ (calculated with the CKD-EPI formula). Out of the 39 participants, 23 were men and 16 were women (Table 1). Form T1 to T2, we observed an ascent in the number of responders (from 17/39 to 34/39, x^2^ = 16.2, *p* < 0.0001).

Five of our patients reported low-grade fever in the 48 h following the first Tozinameran dose, while seven experienced muscle/joint pain or headache. These numbers dropped to 3/39 and 5/39 after the second and 2/39 and 3/39 after the third dose. Swelling and pain at injection site occurred in 10/39, 8/39 and 5/39 of the participants, after the first, second and third dose administration, respectively. Serious side effects, such as anaphylaxis, had not been reported. None of the side effects affected our results.

### 3.2. Alterations in the Concentrations of Cellular Subpopulations

**Figure 1:**
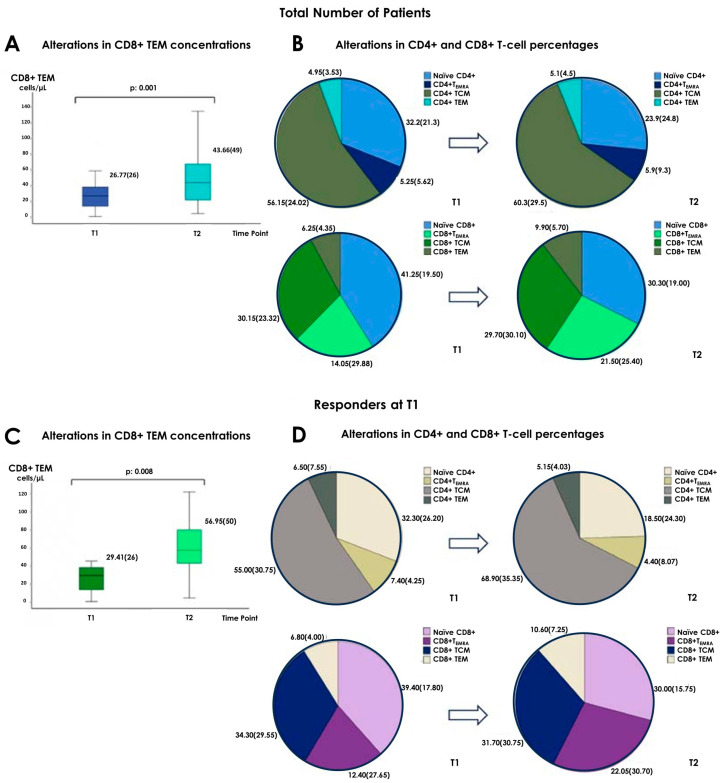
The upper panel depicts changes in the total number of patients; the lower panel illustrates the same changes in “responders at T1”. (**A**) Significant increase in CD8 TEM cells and (**B**) changes in the proportions of CD4 and CD8 subpopulations from T1 to T2 in the total number of patients (**C**) Significant increase in CD8 TEM cells and (**D**) changes in the proportions of CD4 and CD8 subpopulations from T1 to T2 in the “responders at T1”.

In the total number of patients, there was an increase in the concentration of CD8+ TEM from T1 to T2 time points, (from 26.77 (26) cells/µL to 43.66 (49) cells/µL, respectively, *p* = 0.01). Data are presented in Figure 1 and prescribed in Supplementary Table S1.

In the “responders at T1” group, we observed a slightly more prominent ascent in the CD8+ TEM population, with a concurrent elevation of CD8+ T_EMRA_ [29.41 (26) cells/µL at T1 vs. 56.95 (50) cells/µL at T2, *p* = 0.008 and 72.33 (140) cells/µL at T1 vs. 126.15 (168) cells/µL at T2, *p* = 0.022, for CD8+ TEM and CD8+ T_EMRA,_ respectively] (Table 2 and Figure 1).

**Figure 2:**
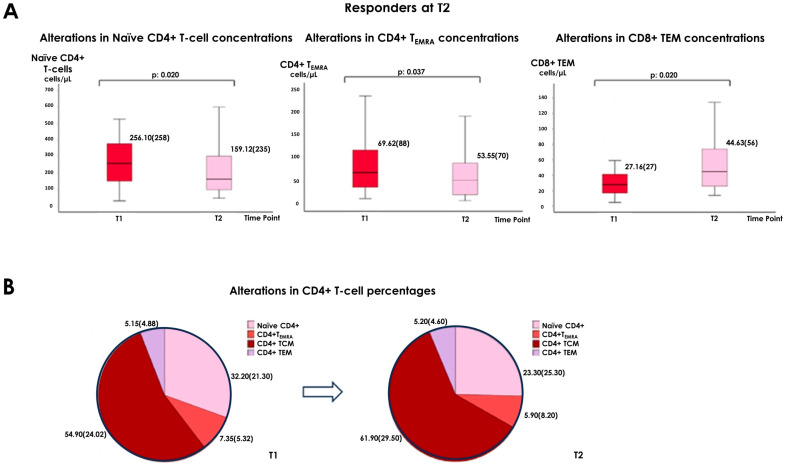
Alterations in T lymphocytes in the “responders at T2” (**A**) significant reduction in CD4 Naïve, CD4 TEMRA and increased in CD8 EM cells from T1 to T2, (**B**) changes in the proportions of CD4 subpopulations in the same group from T1 to T2.

As for “responders at T2”, they displayed a drop of naïve CD4+ [256.10 (258) cells/µL at T1 vs. 159.12 (235) cells/µL at T2, *p* = 0.020] and CD4+ T_EMRA_ [69.62 (88) cells/µL at T1 vs. 53.55 (70) cells/µL at T2, *p* = 0.037] numbers, as well as an increase in the that of CD8+ TEM [27.16 (27) cells/µL at T1 vs. 44.63 (56) cells/µL at T2, *p* = 0.020] (Table 3 and Figure 2).

### 3.3. Alterations in the Percentages of Cellular Subpopulations

As for the observed changes in cellular subset proportions, in the total number of patients there was an ascent in the percentages of CD4+ TCM [56.15 (24.02)% at T1 vs. 60.3 (29.5)% at T2, *p* = 0.016] and CD8+ TEM [6.25 (4.35)% at T1 vs. 9.9 (5.7)% at T2, *p* = 0.042], along with a decrease in that of naïve CD4+ [32.2 (21.3)% at T1 vs. 23.9 (24.8)% at T2, *p* = 0.012] and CD8+ [41.25 (19.50)% at T1 vs. 30.30 (19.00)% at T2, *p* = 0.028] (Supplementary Table S1 and Figure S1).

Among responders at T1, CD4+ TCM [55 (30.75)% at T1 vs. 68.9 (35.35)% at T2, *p* = 0.013] and CD8+ TEM [6.8 (4)% at T1 vs. 10.6 (7.25)% at T2, *p* = 0.044] proportions increased, while there was a drop in that of naïve CD4+ [32.3 (26.2)% vs. 18.5 (24.3)%, *p* = 0.030] (Table 2 and Figure 1). Responders at T2 presented an elevation of CD4+ TCM percentage [54.9 (24.02)% at T1 vs. 61.9 (29.5)% at T2, *p* = 0.004], with a concurrent decrease in the proportion of naïve CD4+ T-cells [32.2 (21.3)% at T1 vs. 23.3 (25.3)% at T2, *p* = 0.006] (Table 3 and Figure 2). Such changes were not evident in the non-responders groups at T1 or T2. (Supplementary Tables S2 and S3)

Interestingly, the concentrations and/or proportions of CD3+PD1+ T-lymphocytes did not change remarkably during the time interval from T1 to T2 neither in the whole cohort of patients, nor in the responders’ and non-responders’ subgroups.

### 3.4. Differences between Responders and Non-Responders at T1 and T2

At T1 we did not observe any statistically significant differences between responders and non-responders with regards to the composition of their cellular subpopulations (Table 4). On the contrary, at T2, responders had more elevated concentrations of CD28-expressing CD8+ T-lymphocytes [227.15 (166) cells/µL vs. 131.44 (121) cells/µL for responders and non-responders, respectively, *p* = 0.036] and lower numbers of CD4+CD28^null^ T-cells [59.65 (66) cells/µL in responders vs. 161.19 (92) cells/µL in non-responders, *p*: 0.026]. They also displayed higher CD3+CD4+CD28+ percentages [93.8 (6)% vs. 72.05 (30.73) in responders and non-responders, respectively, *p* = 0.005]. At the same time point, CD4+CD28^null^ cell proportions were lower among responders [6.1 (5.5)% vs. 20.7 (25)% for responders and non-responders, respectively, *p* = 0.004] (Table 5).

We should underline that no statistically significant differences were recorded between responders and non-responders with regards to CD3+PD1+ T-cell numbers and percentages neither at T1 nor at T2 (Table 3 and Table 4).

### 3.5. Study of the Effects of the Third Tozinameran Dose on Cellular Subpopulations’ Compositions

In order to focus solely on the effects of the third vaccination dose, we separated patients into four groups: (a) responders at T1 that remained responders at T2 (*n* = 14), (b) non-responders at T1 who achieved a response only at T2 (*n* = 20), (c) non-responders at both T1 and T2 (*n* = 2), (d) responders at T1 who were no longer regarded as responders at T2 (*n* = 3).

Since only very few patients were stratified into the third and the fourth group, the relevant measurements were not reliable and are thus not displayed below.

In Table 6 we present the changes in T-cell subpopulation concentrations and percentages from T1 to T2 among patients of the first group.

These individuals had an increase in CD8+ TEM and a reduction in CD4+ T_EMRA_ concentrations [37.03 (22) cells/µL vs. 61.7 (60) cells/µL, *p*: 0.015 and 80.46 (73) cells/µL vs. 43.35 (83) cells/µL, *p*: 0.023, respectively], as well as an elevation of CD4+ TCM and a drop of naïve CD4+ T-cell percentages [54.30 (29.73)% vs. 68.90 (31.45)%, *p* = 0.009 and 30.10 (24.50)% vs. 18.35 (21.28)%, *p* = 0.048]. Patients of the second group did not present any statistically significant alterations in the same time interval (Table 7).

The comparison of cellular subpopulation concentrations and percentages between individuals having been stratified into the first two groups are shown in Table 8 and Table 9, respectively. At T1, higher percentages of CD4+ TEM were recorded among patients of the first group [7.56 (7.45)% vs. 3.85 (3.35)%, *p* = 0.014] and at T2, the same individuals had lower proportions of CD4+CD28+ T-lymphocytes [92.75 (6.92)% vs. 95.85 (6.77)%, *p* = 0.035].

## 4. Discussion

It is widely known that, in most cases, in order to enhance the immune response that is induced by the initial priming dose of a vaccine, repeated administration is required [14]. Vaccination against COVID-19 does not constitute an exception, as anti-SARS-CoV-2 Ab concentrations tend to decline over time and booster shots are necessary for maintaining them at protective levels, especially in patients having received immunosuppression [15,16]. Several studies have focused on the third dose of Tozinameran, proving that it can significantly ameliorate both humoral and anti-SARS-CoV-2-specific T-cell responses among immunocompromised individuals, such as those with hematological or solid organ malignancies and KTRs [17,18]. In accordance with these findings, we observed that, in our sample, the number of responders (defined as those who had developed either protective NAb titers or sufficient anti-spike-specific T-cell activation) significantly increased after the third dose of Tozinameran.

However, the cost–benefit relationship regarding the policy of multiple booster doses administration seems to be an area of controversy, with some authors supporting that it could even reduce immunization effectiveness [11,12,13]. Given all the above, we attempted to examine the potential impact of repetitive SARS-CoV-2 vaccinations with Tozinameran on T-lymphocyte differentiation and exhaustion procedures among KTRs. In the present study, we focused on the effect of the first two booster doses on the previously mentioned phenomena. More specifically, we investigated whether response to these vaccinations could be associated with immunoscenesence or immunoexhaustion. Response after the first and the second booster dose (e.g., the second and the third Tozinameran dose) administration was defined as ability of the patient to develop protective anti-SARS-CoV-2 NAbs and/or a positive ELISpot test at T1 and at T2, respectively.

According to our findings, from T1 to T2, in the total number of patients, there was an ascent in the concentration of CD8+ TEM. Regarding alterations in cellular subpopulations’ percentages, they displayed a clear shift towards more differentiated forms, as we observed a drop in the proportions of naïve CD4+ and CD8+ T-lymphocytes, along with an increase in that of CD4+ TCM and CD8+ TEM. The above changes were more obvious among patients who were considered responders to vaccination at either T1 or T2. More specifically, responders at T1 presented an elevation in the number of CD8+ TEM and T_EMRA_ and in the percentages of CD4+ TCM and CD8+ TEM, as well as a decrease in the proportion of naïve CD4+ T-lymphocytes. As for responders at T2, we recorded an ascent of CD8+ TEM and a reduction in naïve CD4+ and CD4+ T_EMRA_. They also exhibited an increase in the percentage of CD4+ TCM and a decrease in that of naïve CD4+ T-lymphocytes. In addition, although at T1 there were no statistically significant differences among responders and non-responders regarding cellular subpopulations’ composition, at T2 responders had higher levels of CD8+ T-lymphocytes expressing the co-stimulatory factor CD28, as well as greater proportions of CD4+CD28+ and lower numbers and percentages of CD4+CD28− T-cells. In order to “isolate” the effect of the third Tozinameran dose, we performed a comparison between responders at T1 that remained responders at T2 and patients that were regarded as non-responders at T1 and achieved a response only at T2. We found that solely the patients of the first group had an elevation of CD8+ TEM and a drop of CD4+ T_EMRA_ numbers, along with an increase in CD4+ TCM and a reduction in naïve CD4+ T-cell proportions in the time interval from T1 to T2. Additionally, at T1, they displayed higher percentages of CD4+ TEM and at T2, they had lower proportions of CD4+CD28+ T-lymphocytes.

Antigen stimulation induces the production of antigen-specific effector cells that die shortly after peak response, as well as of memory precursors, which finally develop into prolonged-survival memory cells [19]. Based on the expression of the surface molecules CD45RA/RO, CCR7, CD27 and CD28, memory T-cells can be segregated into four subgroups, depending on their differentiation status. More specifically, we have naïve T-cells (CD45RA+, CD45RO−CD27+CD28+CCR7+), TCM (CD45RA-CD45RO+CD27+CD28+CCR7+), TEM (CD45RA−CD45RO+CD27±CD28±CCR7−) and T_EMRA_ (CD45RA+CD45RO−CD27−CD28−CCR7−). During the differentiation process, memory lymphocytes gradually lose the ability to secrete interleukin-2 (IL-2), while they progressively produce an increasing number of effector (including tumor necrosis factor-α -TNFα-, IL-4 and IL-5) and cytotoxic (e.g., perforin, granzymes) molecules [1]. TEM are found in the systemic circulation, while TCM, apart from the blood, reside in lymphoid organs [20].

Senescence is a condition characterized by the loss of cellular proliferation capacity and the shortening of telomeres, with several complex mechanisms mediated by the lack CD27 and CD28 expression and the production of CD57 and KLRG-1 being involved. Based on the above-analyzed cell surface antigen (CD27, CD28, CD57 and KLRG-1) expression pattern, T_EMRA_ are considered par excellence senescent cells, while a proportion of TEM could also be listed as such [1]. However, in the present work, since we had solely utilized CD45RA, CCR7 and CD28 antigens in order to categorize memory T-lymphocytes, apart from CD28^null^, we only classified T_EMRA_ as “aged” cells, so that this group would be more homogenous. Given that condition, we found that response to vaccination is not associated with an increase in senescent (e.g., CD28^null^ or T_EMRA_) cell numbers or percentages.

It has been proven that conditions such as aging and chronic inflammation induce the accumulation of T_EMRA_, while recurrent antigenic stimulation, in addition to promoting differentiation, may also be responsible for the development of irreversible DNA damage and telomere abrasion, thus leading to the loss of T-cell proliferating capacity. Interestingly, T-cell receptor (TCR) ligation favors the expression of the RO isoform of CD45, while the survival of T_EMRA_ and the subsequent increase in their numbers does not necessarily require repeated (infection or vaccination-mediated) antigen exposure [21]. On the contrary, Carrasco et al. demonstrated that, rather than being terminally differentiated, CD8+ T_EMRA_ are actually a heterogenous group of memory cells, with varying expression of granzyme B, Fas ligand (FasL), TNF-α and IFN-γ, with some of them transiently losing CD45RA and producing CCR7 upon antigenic stimulation. In the absence of additional stimuli, these cells re-express CD45RA within a few weeks [22].

The exact impact vaccination has on T-cell differentiation process has not yet been clarified, with some authors even supporting that recurrently stimulated memory cells do not have the exact same phenotype with senescent cells, as they share several surface markers (e.g., CD27, CCR7, KLRG-1, etc.), but also differ in the expression pattern of others (e.g., CD28, CD57) [23].

Based on all the above, the fact that, according to our findings, response to SARS-CoV-2 vaccination with the second and the third Tozinameran dose is not associated with T-cell senescence, could be explained by multiple factors. More specifically, to some degree, this possibly has to do with the definition of senescent cells in our study per se, as in this group we had only included T_EMRA_ and CD28^null^ cells. Had we utilized different surface markers in order to identify senescence, some of the TEM would also be considered “aged”, giving us different results regarding the effect of booster vaccinations to the T-lymphocyte senescence phenomenon. Moreover, based on the findings of Carrasco et al., we could assume that the presence of recurrent antigenic stimulation can partially lead to the predominance of TEM over T_EMRA_, as, due to re-exposure to viral antigens, the latter temporarily lose CD45RA and re-express CCR7. A more delayed examination of cellular populations (with a greater time interval following the third vaccination) would perhaps reveal higher concentrations or percentages of T_EMRA_.

Even more interestingly, when we attempted to compare changes in T-cell subpopulations between patients that had responded to vaccination after the second dose and remained responders post-third dose with those that had initially been non-responders and achieved a response solely after the third dose, we found that only the first ones presented a shift towards more differentiated, non-senescent forms (e.g., an increase in CD8+ TEM and a reduction in CD4+ T_EMRA_ concentrations, with an elevation of CD4+ TCM and a drop of naïve CD4+ T-cell percentages) following the third dose administration. A possible interpretation for the above-mentioned findings could be that these alterations in cellular subpopulations’ compositions indeed accrue as the cumulative result of multiple vaccinations (and the subsequent repeated exposure to an antigenic stimulus) and may not reflect the effect of a single booster dose (in that case, the third Tozinameran dose).

As mentioned previously, immunoexhaustion and immunosenescence are two different dysfunctional states. Exhausted T-cells are in most cases TCM or TEM that have been subjected to chronic or recurrent stimulation. They are characterized by the expression of inhibitory immunoreceptors such as PD-1, LAG-3, TIM-3 and CTLA-4, are unable to secrete cytokines with stimulation and are programmed to go through apoptosis in order to prevent immunopathology due to excessive immune response [1,24]. Whether repeated vaccinations could be responsible for the induction of T-lymphocyte exhaustion is still a matter of controversy. In our work with KTRs, we did not observe an upward trend in the numbers and percentages of PD-1-expressing T-cells that could be attributed the administration of booster Tozinameran doses, neither in the “responders” nor in the “non-responders” group. Moreover, responders had not presented notable differences from non-responders regarding exhausted T-lymphocyte concentrations or percentages in any of the examined time points. On the contrary, Fuentes et al., despite proving that among patients with solid malignancies, those with adequate cellular response had increased percentages of CD8+PD-1+ T-cells, found a negative correlation between PD-1 expression and specific anti-SARS-CoV-2 IFN-γ production [12]. Another approach is that of Wirth et al., who studied the gene expression profile of CD8+ T-lymphocytes following vaccination and concluded that their transcriptome to some degree differs from that of exhausted T-cells.

We are aware that our research has several weak spots. First of all, the way that we defined development of response after the second and the third vaccination with Tozinameran, does not “isolate” the impact of these particular doses on KTRs’ immune system aging process, but rather reflects the cumulative effect of the doses having been administered prior to the examined time point. Consequently, our approach is not optimal when it comes to investigating the interaction of the patients’ immune system with each vaccination separately. To some degree, the drawbacks of this approach are counterpoised by the comparison we performed between responders at T1 that remained responders at T2 and patients that were initially non-responders and achieved a response only at T2. However, a more holistic assessment would also include individuals that were considered non-responders at both T1 and T2, as well as responders at T1 who were no longer responders at T2. In the present study, the relevant results were not reliable due to the small number of participants. A larger cohort of patients would of course allow us to include such information. Moreover, although our initial goal was to include measurements associated with additional booster doses, that was not possible, as, over the course of time, it could result in the involvement of several parameters (e.g., natural exposure to SARS-CoV-2 or other infectious agents) that would complicate our findings. For the same reason, the time intervals between each vaccination and the relevant blood sampling time point were not equal (the interval following the third dose was remarkably shorter than that following the second and that of course affected both the measurements that focused on the cumulative effect of multiple vaccinations and those that examined the results of the third dose only). Although we tried to create a homogenous cohort of patients, that could not be achieved to the absolute extent. Indicatively, there was no uniformity regarding the presence of diabetes mellitus (present in 4/39 participants) or of a negative family history of kidney disease, while the age range of our patients was rather wide. This of course could be corrected in future studies with greater numbers of participants, which would allow us to further segregate them based on such parameters. Another disadvantage is that we did not manage to include a healthy control group. Since the immune system status of healthy individuals at baseline differs a lot from that of KTRs, we believed that using them as controls could possibly complicate our results. However, such an approach could definitely be attempted in future research.

In closing, it should be mentioned that, despite its potential defects, the present study offers an insight into future research directions. Indicatively, an analogous approach could be used in order to examine the effects of multiple administrations of other vaccines (both against the SARS-CoV-2 virus and other infectious agents) on the immunosenescence and immunoexhaustion procedures. The relevant results could be combined in order to give us a complete understanding on how repeated vaccinations may affect these phenomena. Of course, it would be of great interest to compare findings that refer to different vaccine types against COVID-19 (such as mRNA, protein subunit, viral vector vaccines, inactivated or attenuated vaccines). As mentioned before, measurements could be expanded to additional booster doses, even though this would possibly complicate the results due to the involvement of confounding factors. Lastly, another idea would be to compare the results of this study with analogous measurements performed on healthy individuals, or on KTRs who were naturally exposed to SARS-CoV-2 (with all the inaccuracies such a comparison would include).

## 5. Conclusions

In the current literature, there are still insufficient data regarding the potential effects multiple vaccinations could have on the immune system’s senescence and exhaustion procedures, rendering that a matter of controversy. In the present work, we demonstrated that in KTRs, an immunologically distinct group of patients, response to Tozinameran booster doses is not associated with an immunosenescence and/or immunoexhaustion status, although it seems to be connected to the prevalence of more differentiated/activated memory T-cell forms. Of course, to some degree, this finding is affected by the definition of senescence in our research per se. In the same context, it has been proposed that recurrent antigenic stimulation through vaccination could possibly even lead to the conversion of memory T-cells that until recently were being considered terminally differentiated (and consequently unable to proliferate), into more functional forms.

## Figures and Tables

**Table 1:** Clinical parameters of patients at baseline.

Clinical Parameter	MED.(IQR)
Age (years)	47 (16)
eGFR (mL/min/1.73 m^2^)	54.1 (22.7)
Time interval from transplantation (years)	6.9 (14.5)
Duration of potential previous hemodialysis (months)	18 (45)
Tacrolimus levels (ng/mL)	6 (2)
Cyclosporine levels (ng/mL)	256 (186)

**Table 2:** Changes in cellular subpopulation concentrations and percentages from T1 to T2 in the “responders at T1” group.

Cellular Subpopulation	Cell Concentrations (Cells/µL) at Specific Time Points (MED.(IQR))		*p* Value	Cell Percentages (%) at Specific Time Points (MED.(IQR))		*p* Value
	T1	T2		T1	T2	
Naïve CD4+ T-cells	189.87 (193)	125.26 (105)	0.245 (NS)	32.3 (26.2)	18.5 (24.3)	0.030
CD4+ TCM	424.03 (303)	380.42 (411)	0.470 (NS)	55 (30.75)	68.9 (35.35)	0.013
CD4+ TEM	45 (71)	28.83 (40)	0.683 (NS)	7.65 (8.65)	5.1 (3.55)	0.485 (NS)
CD4+ TEMRA	62.75 (70)	22.54 (49)	0.056 (NS)	7.3 (4.38)	4 (5.1)	0.365 (NS)
Naïve CD8+ T-cells	145.19 (172)	132.04 (141)	0.158 (NS)	37.55 (15.38)	30.4 (12.2)	0.052 (NS)
CD8+ TCM	138.12 (138)	155.71 (148)	0.245 (NS)	28.45 (31.95)	34.6 (28.10)	0.423 (NS)
CD8+ TEM	29.41 (26)	56.95 (50)	0.008	6.8 (4)	10.6 (7.25)	0.044
CD8+ TEMRA	72.33 (140)	126.15 (168)	0.022	14.95 (31.85)	16.1 (19.65)	0.088 (NS)
CD3+CD4+CD28+ T-cells	703.54 (412)	520.75 (594)	0.551 (NS)	85.8 (11.87)	92.3 (10.05)	0.717 (NS)
CD3+CD4+CD28− T-cells	78.65 (145)	67.56 (49)	0.470 (NS)	14.15 (12.25)	7.4 (10)	0.642 (NS)
CD3+CD8+CD28+ T-cells	220.73 (165)	176.73 (146)	0.778 (NS)	37.15 (20.8)	36.7 (16.65)	0.897 (NS)
CD3+CD8+CD28− T-cells	221.63 (235)	277.98 (298)	0.594 (NS)	62.75 (18.95)	64 (17.25)	1.0 (NS)
CD3+PD1+ T-cells	41.33 (44)	34.13 (51)	0.826 (NS)	2.8 (3.7)	2.45 (2.6)	0.679 (NS)

In the time interval from T1 to T2, among “responders at T1”, there was an increase in CD8+ TEM and of CD8+ TEMRA concentrations. There was also an elevation of CD4+ TCM and CD8+ TEM percentages and drop in naïve CD4+ T-cell percentages. *p* values < 0.05 are considered statistically significant. NS: non-significant.

**Table 3:** Changes in cellular subpopulation concentrations and percentages from T1 to T2 in the “Responders at T2” group.

Cellular Subpopulation	Cell Concentrations (Cells/µL) at Specific Time Points [MED.(IQR)]		*p* Value	Cell Percentages (%) at Specific Time Points [MED.(IQR)]		*p* Value
	T1	T2		T1	T2	
Naïve CD4+ T-cells	256.10 (258)	159.12 (235)	0.020	32.2 (21.3)	23.3 (25.3)	0.006
CD4+ TCM	436.10 (399)	545.81 (431)	1.0 (NS)	54.9 (24.02)	61.9 (29.5)	0.004
CD4+ TEM	47.92 (55)	31.84 (46)	0.773 (NS)	5.8 (7.55)	4.45 (3.9)	0.991 (NS)
CD4+ TEMRA	69.62 (88)	53.55 (70)	0.037	7.4 (5.17)	3.7 (4.55)	0.299 (NS)
Naïve CD8+ T-cells	206.33 (247)	182.27 (248)	0.230 (NS)	42.9 (23.25)	37.75 (18.9)	0.060 (NS)
CD8+ TCM	134.69 (216)	162.48 (155)	0.581 (NS)	24 (23.95)	34.85 (26.57)	0.837 (NS)
CD8+ TEM	27.16 (27)	44.63 (56)	0.020	6.4 (5.85)	9.65 (6.23)	0.071 (NS)
CD8+ TEMRA	93.94 (141)	91.59 (121)	0.361 (NS)	19.5 (29.57)	14.9 (13.45)	0.336 (NS)
CD3+CD4+CD28+ T-cells	753.93 (419)	743.56 (587)	0.845 (NS)	90.9 (14.63)	92.9 (5.47)	0.340 (NS)
CD3+CD4+CD28− T-cells	63.31 (179)	63.23 (54)	0.289 (NS)	9.35 (15)	7.1 (5.73)	0.369 (NS)
CD3+CD8+CD28+ T-cells	214.87 (201)	227.15 (147)	0.558 (NS)	37.15 (29.52)	38.55 (29)	0.531 (NS)
CD3+CD8+CD28− T-cells	284.14 (318)	256.06 (233)	0.453 (NS)	62.75 (28.03)	63 (30.4)	0.513 (NS)
CD3+PD1+ T-cells	41.33 (50)	32.95 (48)	0.558 (NS)	2.65 (2.4)	2.45 (2.2)	0.822 (NS)

In the time interval from T1 to T2, among “responders at T2”, there was a reduction in naïve CD4+ and CD4+ TEMRA concentrations and an increase in the that of CD8+ TEM. Moreover, these patients displayed an elevation of CD4+ TCM and a decrease in naïve CD4+ T-cell percentages. *p* values < 0.05 are considered statistically significant. NS: non-significant.

**Table 4:** Differences in cellular subpopulation concentrations and percentages between responders and non-responders at T1.

Cellular Subpopulation	Cell Concentrations (Cells/µL) at T1 MED.(IQR)			Cell Percentages (%) at T1 MED.(IQR)		
	Responders	Non-Responders	*p* Value	Responders	Non-Responders	*p* Value
Naïve CD4+ T-cells	198.76 (254)	250.25 (224)	0.590 (NS)	32.3 (26.2)	32.1 (20.4)	0.876 (NS)
CD4+ TCM	372.6 (204)	531.54 (441)	0.184 (NS)	55 (30.75)	57.3 (25.1)	0.397 (NS)
CD4+ TEM	42.59 (56)	35.94 (46)	0.318 (NS)	6.5 (7.55)	3.8 (3.3)	0.066 (NS)
CD4+ TEMRA	69.62 (77)	47.75 (81)	0.909 (NS)	7.4 (4.25)	6.7 (6)	0.639 (NS)
Naïve CD8+ T-cells	154.72 (166)	201.85 (255)	0.782 (NS)	39.4 (17.8)	45.1 (19.3)	0.196 (NS)
CD8+ TCM	141.56 (165)	138.03 (211)	0.195 (NS)	34.3 (29.55)	28.3 (26.5)	0.346 (NS)
CD8+ TEM	30.24 (27)	17.7 (28)	0.988 (NS)	6.8 (4)	4.2 (4.8)	0.138 (NS)
CD8+ TEMRA	70.05 (162)	96.12 (115)	0.935 (NS)	12.4 (27.65)	18.8 (31)	0.639 (NS)
CD3+CD4+CD28+ T-cells	742.66 (489)	843.4 (501)	0.232 (NS)	87.8 (13.5)	93.5 (9.4)	0.073 (NS)
CD3+CD4+CD28− T-cells	70.18 (165)	54.67 (142)	0.463 (NS)	12.5 (13.7)	5.9 (10.7)	0.052 (NS)
CD3+CD8+CD28+ T-cells	209.3 (170)	209.37 (235)	0.988 (NS)	37.3 (17.1)	41.9 (32.3)	0.931 (NS)
CD3+CD8+CD28− T-cells	228.62 (221)	226.21 (323)	0.798 (NS)	63.4 (15.4)	59.9 (31.55)	0.931 (NS)
CD3+PD1+ T-cells	41.33 (44)	32.49 (41)	0.322 (NS)	2.8 (3.7)	2.45 (2)	0.319 (NS)

At T1 we did not observe any statistically significant differences between responders and non-responders regarding the composition of their cellular subpopulations. *p* values < 0.05 are considered statistically significant. NS: non-significant.

**Table 5:** Differences in cellular subpopulation concentrations and percentages between responders and non-responders at T2.

Cellular Subpopulation	Cell Concentrations (Cells/µL) at T2 MED.(IQR)			Cell Percentages (%) at T2 MED.(IQR)		
	Responders	Non-Responders	*p* Value	Responders	Non-Responders	*p* Value
Naïve CD4+ T-cells	165.19 (252)	251.75 (405)	0.680 (NS)	23.3 (25.3)	39.7 (27.28)	0.160 (NS)
CD4+ TCM	530.84 (375)	394.16 (443)	0.457 (NS)	61.9 (29.5)	46.95 (40.3)	0.352 (NS)
CD4+ TEM	36.54 (51)	16.35 (73)	0.151 (NS)	5.2 (4.6)	3.6 (3.75)	0.352 (NS)
CD4+ TEMRA	54.54 (78)	44.78 (183)	1.0 (NS)	5.9 (8.2)	9.65 (16)	0.635 (NS)
Naïve CD8+ T-cells	144.55 (156)	159.03 (149)	0.763 (NS)	29.9 (19.2)	34.5 (12.48)	0.467 (NS)
CD8+ TCM	153.18 (160)	214.44 (176)	0.640 (NS)	29.5 (28.1)	41.6 (27.4)	0.467 (NS)
CD8+ TEM	43.66 (55)	31.5 (71)	0.424 (NS)	9.9 (5.6)	9 (9.35)	0.499 (NS)
CD8+ TEMRA	123.01 (142)	42.26 (80)	0.094 (NS)	22.7 (25.1)	15.7 (26.87)	0.407 (NS)
CD3+CD4+CD28+ T-cells	756.34 (368)	446.57 (925)	0.239 (NS)	93.8 (6)	72.05 (30.73)	0.005
CD3+CD4+CD28− T-cells	59.65 (66)	161.19 (92)	0.026	6.1 (5.5)	20.7 (25)	0.004
CD3+CD8+CD28+ T-cells	227.15 (166)	131.44 (121)	0.036	40 (30.4)	31.55 (15.77)	0.154 (NS)
CD3+CD8+CD28− T-cells	256.06 (309)	327.8 (343)	0.979 (NS)	60 (30.9)	68.4 (12.85)	0.127 (NS)
CD3+PD1+ T-cells	32.95 (48)	59.85 (72)	0.624 (NS)	2.45 (2.2)	3.55 (2.2)	0.269 (NS)

At T2, responders displayed more elevated concentrations of CD3+CD8+CD28+ and lower numbers of CD3+CD4+CD28− T-cells. They also had higher CD3+CD4+CD28+ and lower CD3+CD4+CD28− T-cell proportions. *p* values < 0.05 are considered statistically significant. NS: non-significant.

**Table 6:** Changes in T-cell subpopulation concentrations and percentages from T1 to T2 among responders at T1 that remained responders at T2.

Cellular Subpopulation	Cell Concentrations (Cells/µL) at Specific Time Points [MED.(IQR)]		*p* Value	Cell Percentages (%) at Specific Time Points [MED.(IQR)]		*p* Value
	T1	T2		T1	T2	
Naïve CD4+ T-cells	251.67 (280)	138.80 (213)	0.158	30.10 (24.50)	18.35 (21.28)	0.048
CD4+ TCM	432.58 (242)	465.76 (467)	0.530	54.30 (29.73)	68.90 (31.45)	0.009
CD4+ TEM	53.86 (64)	42.2 (59)	0.754	7.65 (7.45)	5.35 (4.90)	0.572
CD4+ TEMRA	80.46 (73)	43.35 (83)	0.023	7.45 (3.58)	4.40 (7.62)	0.187
Naïve CD8+ T-cells	175.81 (198)	144.55 (129)	0.239	38.40 (20.27)	29 (18.27)	0.116
CD8+ TCM	138.12 (178)	132.44 (144)	0.239	34.25 (30.95)	31.7 (26.78)	0.802
CD8+ TEM	37.03 (22)	61.7 (60)	0.015	7.2 (4.15)	10.60 (8.45)	0.109
CD8+ TEMRA	80.43 (202)	177.5 (192)	0.050	12.75 (29.75)	22.05 (34.28)	0.221
CD3+CD4+CD28+ T-cells	774.71 (462)	795.42 (495)	0.754	87.8 (14.12)	92.75 (6.92)	0.594
CD3+CD4+CD28− T-cells	118 (181)	66.18 (51)	0.308	11.75 (14.85)	7.10 (6.50)	0.510
CD3+CD8+CD28+ T-cells	223.53 (186)	198.18 (171)	0.583	37.15 (16.05)	36.15 (29.08)	0.925
CD3+CD8+CD28− T-cells	330.91 (221)	320.73 (287)	0.875	63.6 (14.65)	64.65 (29.55)	0.975
CD3+PD1+ T-cells	44.72 (63)	38.86 (50)	0.814	2.8 (5.4)	2.45 (2.8)	0.510

Responders at T1 who remained responders at T2 had an increase in CD8+ TEM and a reduction in CD4+ TEMRA concentrations, as well as an elevation of CD4+ TCM and a drop of naïve CD4+ T-cell percentages.

**Table 7:** Changes in T-cell subpopulation concentrations and percentages from T1 to T2 among non-responders at T1 who achieved response only at T2.

Cellular Subpopulation	Cell Concentrations (Cells/µL) at Specific Time Points [MED.(IQR)]		*p* Value	Cell Percentages (%) at Specific Time Points [MED.(IQR)]		*p* Value
	T1	T2		T1	T2	
Naïve CD4+ T-cells	265.10 (222)	202.77 (293)	0.061	32.4 (21.98)	25.10 (26.63)	0.053
CD4+ TCM	553.26 (427)	542.61 (317)	0.532	56.05 (25.53)	58.35 (31.20)	0.191
CD4+ TEM	36.96 (45)	33.11 (46)	0.910	3.85 (3.35)	4.45 (4.72)	0.478
CD4+ TEMRA	47.78 (81)	57.94 (75)	0.307	6.8 (6.67)	7.25 (9.95)	0.955
Naïve CD8+ T-cells	205.41 (270)	134.04 (259)	0.570	44.9 (22.27)	31.65 (26.78)	0.191
CD8+ TCM	141.81 (229)	158.31 (167)	0.776	27.4 (22.10)	26.7 (30.10)	0.712
CD8+ TEM	18.47 (32)	30.05 (28)	0.394	3.75 (4.9)	8.9 (7.9)	0.379
CD8+ TEMRA	92.29 (120)	108.23 (94)	0.733	19.4 (30.30)	22.10 (23.55)	0.650
CD3+CD4+CD28+ T-cells	840.86 (389)	743.56 (464)	0.983	93.7 (7.72)	95.85 (6.77)	0.433
CD3+CD4+CD28− T-cells	52.83 (162)	46.67 (79)	0.647	5.25 (9.15)	4.65 (6.13)	0.557
CD3+CD8+CD28+ T-cells	212.18 (268)	245.69 (184)	0.231	42.45 (30.10)	49.25 (31.07)	0.327
CD3+CD8+CD28− T-cells	275.31 (399)	210.79 (239)	0.446	58.70 (29.72)	50.15 (30.78)	0.349
CD3+PD1+ T-cells	30.28 (46)	31.73 (28)	0.500	2.15 (2)	2.4 (2)	0.879

Non-responders at T1 who achieved response only at T2 did not present any statistically significant alterations in T-cell subpopulation concentrations and percentages in the time interval from T1 to T2.

**Table 8:** Comparison of cellular subpopulation concentrations and percentages at the T1 time point, between responders at T1 that remained responders at T2 and non-responders at T1 who achieved response only at T2.

Cellular Subpopulation	Cell Concentrations (Cells/µL) at T1 MED.(IQR)			Cell Percentages (%) at T1 MED.(IQR)		
	Responders at Both T1 and T2	Non Responders at T1—Responders at T2	*p* Value	Responders at Both T1 and T2	Non Responders at T1—Responders at T2	*p* Value
Naïve CD4+ cells	251.67 (280)	265.10 (222)	0.891	30.10 (24.50)	32.4 (21.98)	0.722
CD4+ TCM	432.58 (242)	553.26 (427)	0.262	54.30 (29.73)	56.05 (25.53)	0.377
CD4+ TEM	53.86 (64)	36.96 (45)	0.092	7.65 (7.45)	3.85 (3.35)	0.014
CD4+ TEMRA	80.46 (73)	47.78 (81)	0.444	7.45 (3.58)	6.8 (6.67)	0.357
Naïve CD8+ T-cells	175.81 (198)	205.41 (270)	0.570	38.40 (20.27)	44.9 (22.27)	0.135
CD8+ TCM	138.12 (178)	141.81 (229)	0.860	34.25 (30.95)	27.4 (22.10)	0.442
CD8+ TEM	37.03 (22)	18.47 (32)	0.092	7.2 (4.15)	3.75 (4.9)	0.054
CD8+ TEMRA	80.43 (202)	92.29 (120)	0.984	12.75 (29.75)	19.4 (30.30)	0.896
CD3+CD4+CD28+ T-cells	774.71 (462)	840.86 (389)	0.512	87.8 (14.12)	93.7 (7.72)	0.084
CD3+CD4+CD28− T-cells	118 (181)	52.83 (162)	0.180	11.75 (14.85)	5.25 (9.15)	0.050
CD3+CD8+CD28+ T-cells	223.53 (186)	212.18 (268)	0.925	37.15 (16.05)	42.45 (30.10)	0.760
CD3+CD8+CD28− T-cells	330.91 (221)	275.31 (399)	0.488	63.6 (14.65)	58.70 (29.72)	0.679
CD3+PD1+ T-cells	44.72 (63)	30.28 (46)	0.152	2.8 (5.4)	2.15 (2)	0.231

At the T1 time point, responders at T1 that remained responders at T2 had higher percentages of CD4+ TEM.

**Table 9:** Comparison of cellular subpopulation concentrations and percentages at the T2 time point, between responders at T1 that remained responders at T2 and non-responders at T1 who achieved response only at T2.

Cellular Subpopulation	Cell Concentrations (Cells/µL) at T2 MED.(IQR)			Cell Percentages (%) at T2 MED.(IQR)		
	Responders at Both T1 and T2	Non Responders at T1—Responders at T2	*p* Value	Responders at Both T1 and T2	Non Responders at T1—Responders at T2	*p* Value
Naïve CD4+ cells	138.80 (213)	202.77 (293)	0.280	18.35 (21.28)	25.10 (26.63)	0.316
CD4+ TCM	465.76 (467)	542.61 (317)	0.909	68.90 (31.45)	58.35 (31.20)	0.799
CD4+ TEM	42.2 (59)	33.11 (46)	0.537	5.35 (4.90)	4.45 (4.72)	0.468
CD4+ TEMRA	43.35 (83)	57.94 (75)	0.423	4.40 (7.62)	7.25 (9.95)	0.444
Naïve CD8+ T-cells	144.55 (129)	134.04 (259)	0.909	29 (18.27)	31.65 (26.78)	0.421
CD8+ TCM	132.44 (144)	158.31 (167)	0.945	31.7 (26.78)	26.7 (30.10)	0.769
CD8+ TEM	61.7 (60)	30.05 (28)	0.059	10.60 (8.45)	8.9 (7.9)	0.316
CD8+ TEMRA	177.5 (192)	108.23 (94)	0.347	22.05 (34.28)	22.10 (23.55)	0.953
CD3+CD4+CD28+ T-cells	795.42 (495)	743.56 (464)	0.723	92.75 (6.92)	95.85 (6.77)	0.035
CD3+CD4+CD28− T-cells	66.18 (51)	46.67 (79)	0.200	7.10 (6.50)	4.65 (6.13)	0.055
CD3+CD8+CD28+ T-cells	198.18 (171)	245.69 (184)	0.465	36.15 (29.08)	49.25 (31.07)	0.321
CD3+CD8+CD28− T-cells	320.73 (287)	210.79 (239)	0.723	64.65 (29.55)	50.15 (30.78)	0.358
CD3+PD1+ T-cells	38.86 (50)	31.73 (28)	0.950	2.45 (2.8)	2.4 (2)	0.722

At the T2 time point, responders at T1 that remained responders at T2 had lower proportions of CD3+CD4+CD28+ T-cells.

## Data Availability

All data are available upon request.

## Supplementary Materials

The following supporting information can be downloaded at: https://www.mdpi.com/article/10.3390/vaccines12080877/s1, Figure S1: Gating strategy of CD45+, CD3+, CD4+, CD8+, CD45RA+, CCR7+ and CD31+ cells, defining Naïve, TCM, TEM and T_EMRA_, and RTE.; Figure S2: Gating strategy for CD45+, CD3+, CD28+ and PD1+ cells.; Table S1: Changes in cellular subpopulation concentrations and percentages from T1 to T2 in the total number of patients; Table S2: Changes in cellular subpopulation concentrations and percentages from T1 to T2 in non-responders to T1; Table S3: Changes in cellular subpopulation concentrations and percentages from T1 to T2 in non-responders to T2.
