# Supplementary material for: Vaccination with Tozimameran Induces T-Cell Activation, but Not Senescent or Exhaustive Alterations, in Kidney Transplant Recipients
Source: Vaccines (Basel). 2024 Aug 2;12(8):877. doi: 10.3390/vaccines12080877 (PMC11360383; doi:10.3390/vaccines12080877)
Supplement: Supplementary file 1 [file vaccines-12-00877-s001.zip › vaccines-3047954-supplementary.pdf]

Supplement material

Supplement Figure S1. Gating strategy of CD45+, CD3+, CD4+, CD8+, CD45RA+, CCR7+ and CD31+ cells, defining Naïve, TCM, TEM and T_{EMRA}, and RTE. Cell compartments in red squares are T_{EMRA} (senescent cells) and those in blue squares are RTE, CD4 and CD8, respectively

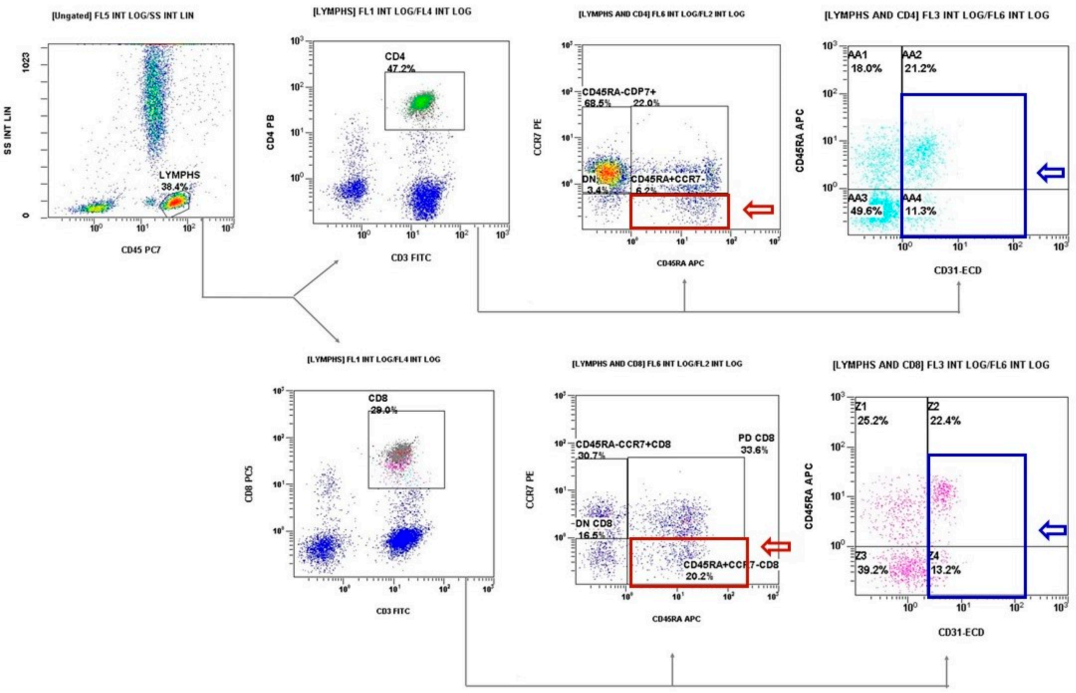

Suppl. Figure S2. Gating strategy for CD45+, CD3+, CD28+ and PD1+ cells. CD28- cells, defined are senescent, are encircled in red square, while PD1+, exhausted, are encircled in the green square.

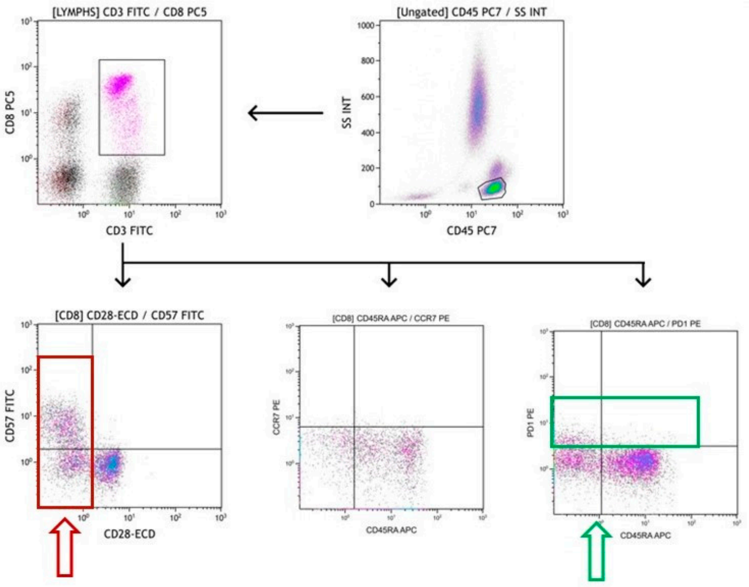

Supplement Table S1. Changes in cellular subpopulation concentrations and percentages from T1 to T2 in the total number of patients

Cellular Subpopulation	Cell concentrations (cells/µL) at specific time points [MED.(IQR)]		p	Cell percentages (%) at specific time points [MED.(IQR)]		p
	T1	T2		T1	T2	
Naïve CD4+ T-cells	219.51(187)	146.96(333)	0.075 (NS)	32.2(21.3)	23.9(24.8)	0.012
CD4+ TCM	372.6(309)	530.84(346)	0.797 (NS)	56.15(24.02)	60.3(29.5)	0.016
CD4+ TEM	35.28(50)	29.94(47)	0.734 (NS)	5.4(7.53)	3.9(3.83)	0.925 (NS)
CD4+ TEMRA	55.87(65)	33.78(45)	0.063 (NS)	7.25(5.33)	3.8(4.92)	0.416 (NS)
Naïve CD8+ T-cells	196.9(209)	172.33(175)	0.245 (NS)	41.25(19.50)	30.30(19.00)	0.028
CD8+ TCM	134.69(183)	169.28(145)	0.829 (NS)	27.4(28.28)	36(24.75)	0.940 (NS)
CD8+ TEM	26.77(26)	43.66(49)	0.01	6.25(4.35)	9.9(5.7)	0.042
CD8+ TEMRA	74.61(122)	72.52(105)	0.245 (NS)	17.8(29.35)	14.9(13.45)	0.184 (NS)
CD3+CD4+CD28+ T-cells	664.42(459)	728.02(653)	0.533 (NS)	88.65(14.75)	92.3(7.33)	0.599 (NS)
CD3+CD4+CD28- T-cells	70.18(157)	66.18(81)	0.602 (NS)	11.2(15.28)	7.65(7.7)	0.556 (NS)
CD3+CD8+CD28+ T-cells	209.3(177)	198.18(182)	0.326 (NS)	37.15(30.10)	37.05(28.65)	0.388 (NS)
CD3+CD8+CD28- T-cells	228.62(296)	267.03(235)	0.144 (NS)	62.75(27.92)	63.95(29.43)	0.409 (NS)
CD3+PD1+ T-cells	39.52(47)	35.55(50)	0.966 (NS)	2.7(2.4)	2.6(2.2)	0.869 (NS)

In the time interval from T1 to T2, in the total number of patients, there was an increase in the concentration of CD8+ TEM, as well as an ascent in the percentages of CD4+ TCM and CD8+ TEM and a decrease in the proportions of naïve CD4+ and CD8+ T-cells

p values < 0.05 are considered statistically significant

*NS: non-significant

Supplement Table S2. Changes in cellular subpopulation concentrations and percentages from T1 to T2 in non-responders to T1

**Cellular Subpopulation**	**Cell concentrations (cells/µL) at specific time points [MED.(IQR)]**		**p**	**Cell percentages (%) at specific time points [MED.(IQR)]**		**p**
	T1	T2		T1	T2	
Naïve CD4+ T-cells	244.40(189)	193.73(420)	0.163 (NS)	34.05(25.7)	24.1(29.6)	0.162 (NS)
CD4+ TCM	344.54(374)	603.05(330)	0.717 (NS)	50.5(25.82)	67.9(31.2)	0.379 (NS)

CD4+ TEM	24.25(41)	30.82(54)	0.836 (NS)	3.6(3.28)	3.8(4.45)	0.587 (NS)
CD4+ TEMRA	47.78(63)	46.89(57)	0.352 (NS)	7.1(7.48)	3.6(5.25)	0.877 (NS)
Naïve CD8+ T-cells	239.86(334)	206.94(275)	0.717 (NS)	47.35(14)	41.6(21.9)	0.179 (NS)
CD8+ TCM	134.24(266)	173.85(141)	0.535 (NS)	27.4(23.85)	36.9(21.6)	0.623 (NS)
CD8+ TEM	16.49(25)	31.54(52)	0.352 (NS)	3.2(4.3)	7.5(10.4)	0.393 (NS)
CD8+ TEMRA	93.94(108)	103.08(103)	0.796 (NS)	19.4(27.85)	13.2(12.05)	0.642 (NS)
CD3+CD4+CD28+ T-cells	628.26(488)	751.11(514)	0.627 (NS)	94.5(16.5)	92.3(8.25)	0.695 (NS)
CD3+CD4+CD28- T-cells	46.07(121)	64(122)	0.970 (NS)	5.7(16.98)	7.7(8.10)	0.695 (NS)
CD3+CD8+CD28+ T-cells	198.76(269)	245.16(187)	0.108 (NS)	34.4(36.25)	39.7(34.4)	0.232 (NS)
CD3+CD8+CD28- T-cells	275.31(615)	256.05(227)	0.179 (NS)	67.05(36.67)	62.3(33.75)	0.218 (NS)
CD3+PD1+ T-cells	32.49(41)	35.55(52)	1.0 (NS)	2.45(2)	2.75(2.2)	0.641 (NS)

In the time interval from T1 to T2, among the "non-responders at T1", there were no statistically significant changes in the numbers and percentages of any po the examined cellular subpopulation
p values < 0.05 are considered statistically significant

*NS: non-significant

Supplement Table S3. Changes in cellular subpopulation concentrations and percentages from T1 to T2 in non-responders to T2

Cellular Subpopulation	Cell concentrations (cells/μL) at specific time points [MED.(IQR)]		p	Cell percentages (%) at specific time points [MED.(IQR)]		p
	T1	T2		T1	T2	
Naïve CD4+ T-cells	125.92(100)	251.75(405)	0.109 (NS)	32.65(35.42)	39.7(27.28)	0.593 (NS)
CD4+ TCM	310.51(252)	394.16(443)	0.109 (NS)	60.45(34.13)	46.95(40.3)	0.285 (NS)
CD4+ TEM	12.65(23)	16.35(73)	0.593 (NS)	2.55(4.18)	3.6(3.75)	0.593 (NS)
CD4+ TEMRA	17.44(10)	44.78(183)	0.285 (NS)	4.3(2.98)	9.65(16)	1.0 (NS)
Naïve CD8+ T-cells	110.58(105)	159.03(149)	1.0 (NS)	43.7(25.58)	34.5(12.48)	0.109 (NS)

CD8+ TCM	109.36(152)	214.44(176)	0.285 (NS)	39.05(24.15)	41.6(27.4)	0.285 (NS)
CD8+ TEM	11.41(17)	31.50(71)	0.109 (NS)	4.4(3.38)	9(9.35)	0.285 (NS)
CD8+ TEMRA	15.86(49)	42.26(80)	0.109 (NS)	6.2(10)	15.7(26.87)	0.109 (NS)
CD3+CD4+CD28+ T-cells	381.93(319)	446.57(925)	0.068 (NS)	80.25(23.77)	72.05(30.73)	0.465 (NS)
CD3+CD4+CD28- T-cells	78.65(41)	161.19(92)	0.144 (NS)	19.4(24.03)	20.7(25)	0.465 (NS)
CD3+CD8+CD28+ T-cells	97.04(201)	131.44(121)	0.144 (NS)	33.3(39.63)	31.55(15.77)	0.465 (NS)
CD3+CD8+CD28- T-cells	157.05(119)	327.8(343)	0.068 (NS)	64.75(38.23)	68.4(12.85)	0.465 (NS)
CD3+PD1+ T-cells	32.82(40)	59.85(72)	0.068 (NS)	2.9(2.1)	3.55(2.2)	0.066 (NS)

In the time interval from T1 to T2, among the “non-responders at T2”, there were no statistically significant changes in the numbers and percentages of any po the examined cellular subpopulation
p values < 0.05 are considered statistically significant

*NS: non-significant
